# Super-Resolution of Plant Disease Images for the Acceleration of Image-based Phenotyping and Vigor Diagnosis in Agriculture

**DOI:** 10.3390/s17112557

**Published:** 2017-11-06

**Authors:** Kyosuke Yamamoto, Takashi Togami, Norio Yamaguchi

**Affiliations:** PS Solutions Corp., 1-5-2 Higashi-Shimbashi, Minato-ku, Tokyo 105-7104, Japan; takashi.togami@g.softbank.co.jp (T.T.); norio.yamaguchi2@g.softbank.co.jp (N.Y.)

**Keywords:** super-resolution, deep learning, convolutional neural network, disease classification, agriculture

## Abstract

Unmanned aerial vehicles (UAVs or drones) are a very promising branch of technology, and they have been utilized in agriculture—in cooperation with image processing technologies—for phenotyping and vigor diagnosis. One of the problems in the utilization of UAVs for agricultural purposes is the limitation in flight time. It is necessary to fly at a high altitude to capture the maximum number of plants in the limited time available, but this reduces the spatial resolution of the captured images. In this study, we applied a super-resolution method to the low-resolution images of tomato diseases to recover detailed appearances, such as lesions on plant organs. We also conducted disease classification using high-resolution, low-resolution, and super-resolution images to evaluate the effectiveness of super-resolution methods in disease classification. Our results indicated that the super-resolution method outperformed conventional image scaling methods in spatial resolution enhancement of tomato disease images. The results of disease classification showed that the accuracy attained was also better by a large margin with super-resolution images than with low-resolution images. These results indicated that our approach not only recovered the information lost in low-resolution images, but also exerted a beneficial influence on further image analysis. The proposed approach will accelerate image-based phenotyping and vigor diagnosis in the field, because it not only saves time to capture images of a crop in a cultivation field but also secures the accuracy of these images for further analysis.

## 1. Introduction

Image analysis approaches have been used for various purposes in agriculture such as vigor diagnosis and phenotyping. Until several years ago, fixed cameras were used to monitor a few selected plants in a time-series. Some recent studies have attempted to detect tomato fruits including immature ones [1], to measure the internode length of tomato seedlings [2], to estimate yield in an apple orchard [3], to detect citrus fruit [4] and immature peach fruit [5], to measure the vegetation index of wheat [6], and to detect the flowering timing of paddy rice [7] under actual cultivation conditions. These studies have developed many algorithms of image analysis to capture vigor information and plant phenotypes.

One of the most innovative technologies in recent years is unmanned aerial vehicles (UAVs or drones), which provide an opportunity to rapidly capture numerous plant images in a field. The algorithms of image analysis are applied to the images captured by UAVs to assess the occurrence of potato disease [8], to map vegetation fraction in wheat fields [9], to detect weeds [10], to estimate leaf area index (LAI) [11], to monitor soil erosion [12] and water stress of crops [13], and to measure the height of sorghum plant [14,15].

Although the UAV is a very promising branch of technology, there is a major limitation to its utilization in agriculture: flight time is very limited because of high power consumption. For instance, Inspire 2 (DJI, Guangdong, China), which is the model for professional use, can fly only 25–27 min at a speed of 94 km/h. To capture as many plants as possible, a UAV needs to fly at a high altitude, but the captured image will have a low spatial resolution. According to Torres-Sánchez et al. [10], who compared the image captured at flight altitudes of 30 m with that of 100 m, area coverage increased from 0.16 ha to 1.76 ha, but spatial resolution decreased from 1.14 cm/pixel to 3.81 cm/pixel. In general, the higher resolution an image has, the more information it conveys. Therefore, the capturing of an image at a high altitude makes it difficult to detect features on plants, such as individual organs and lesion. According to Peña et al. [16], the ability of weed discrimination was significantly affected by spatial resolution.

One of the ways to solve the problem is super-resolution methods that aim at producing a detailed and spatial resolution-enhanced image from one or more low-resolution images [17]. The super-resolution methods using multiple and single images have been called multi-frame and single-frame super-resolution methods, respectively [18]. The former reconstructs spatial resolution from multiple images based on sub-pixel shifts or different parameter information, while the latter learns the relationship between low-resolution and high-resolution images using machine learning and conducts the reconstruction [17].

Super-resolution convolutional neural network (SRCNN) [19] is one of the single-frame methods. In the SRCNN, a deep convolutional neural network is used to describe the relation between low-resolution and high-resolution images. The input of the neural network is a low-resolution image, whereas the output is the high-resolution image. According to Dong et al. [19], SRCNN produced better performance than state-of-the-art methods such as the sparse coding method [20], neighbor and linear embedding method [21], sparse coding using the K-SVD method [22,23], and the anchored neighborhood regression method [24].

In agriculture, several studies utilizing super-resolution methods have been conducted to date. Haris et al. [25] investigated the improvement of 3D reconstruction from RGB images taken by UAVs using super-resolution methods. According to their results, measurement errors of width and height of target objects were minimized by super-resolution methods. This results indicated that the application of super-resolution methods improve measurements of crop parameters, such as plant height and leaf area index, using UAVs. Kasturiwala and Aladhake [26] investigated resolution enhancements of infected leaf images. They demonstrated that super-resolution methods improved the fineness of leaf appearances qualitatively and quantitatively. Although these previous studies have demonstrated the potential of super-resolution methods for agricultural purposes, its effectiveness for solving practical problems in agriculture was not evaluated.

This research aimed to evaluate the performance of super-resolution methods in agricultural applications, especially plant disease classification. We used images from a crop disease image dataset [27], which includes images of several crops, such as tomatoes, grapes, apples, corn, and soybeans. We chose the tomato as the target crop, because it holds the largest number of images in the dataset. Super-resolution and conventional image scaling methods were applied to the disease images to increase the spatial resolution. We then conducted the classification of the disease images generated by different methods to evaluate the effectiveness of the super-resolution method for crop disease classification.

## 2. Materials and Methods

### 2.1. Crop Disease Image Dataset

**Figure A1:**
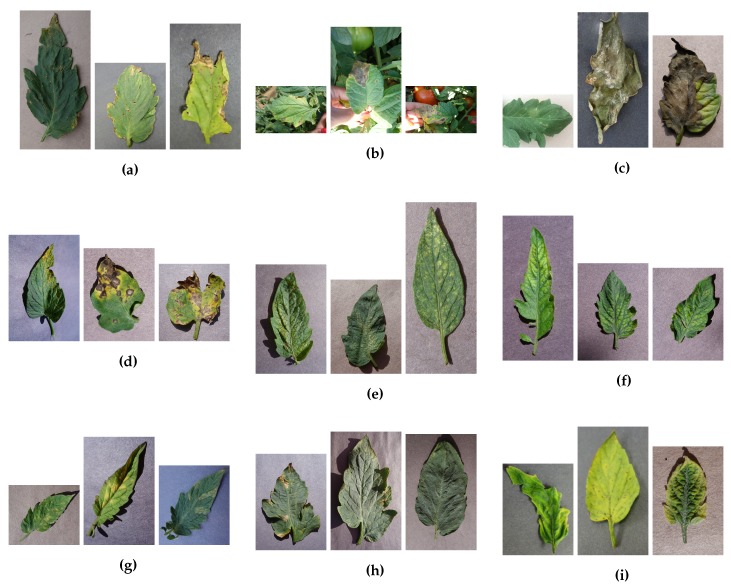
Images of examples of tomato diseases in the Plant Village dataset. (**a**) Xanthomonas campestris pv. Vesicatoria; (**b**) *Alternaria solani*; (**c**) *Phytophthora infestans*; (**d**) *Septoria lycopersici*; (**e**) *Tetranychus urticae*; (**f**) *Tomato mosaic virus*; (**g**) *Fulvia fulva*; (**h**) *Corynespora cassiicola*; (**i**) *Tomato yellow leaf curl virus*.

Crop disease images were obtained from Plant Village dataset [27]. The dataset includes over 50,000 images of 14 crops, such as tomatoes, grapes, apples, corn, and soybeans. We chose tomatoes as our target crop, because tomatoes have the largest number of images (18,149 images) in the dataset. Images of examples of tomato diseases are shown in Figure A1. There are 9 kinds of tomato disease images in the dataset: Xanthomonas campestris pv. Vesicatoria, *Alternaria solani*, *Phytophthora infestans*, *Septoria lycopersici*, *Tetranychus urticae*, Tomato mosaic virus, *Fulvia fulva*, *Corynespora cassiicola*, and *Tomato yellow leaf curl virus*. The number of images of each disease is shown in Table 1.

### 2.2. Performance Evaluation of Super-Resolution

The images were split randomly into two separate sets of images, 80% for training, and the rest for testing. All images were rescaled to 64 × 64 pixels for computational efficiency.

In the training step, we generated low-resolution images by downsampling and blurring the high-resolution images in the training set, and then enlarging them with magnification scales 2–6 using the bicubic method. We then extracted patches of 32 × 32 pixels from the high-resolution and low-resolution images with a stride of 8 pixels; hence, about 362,975 patches were generated for training at each magnification scale. 10% of the patches were used for validation. Input and output of SRCNN were the patches extracted from the low-resolution and high-resolution images, respectively. We trained SRCNN for 50 epochs with mini batches of size 64.

In the testing step, we generated the low-resolution images by the same method as that used in the training step. We then applied the SRCNN model generated in the training step to produce the super-resolution images. For the comparison, we also generated low-resolution images using 5 different conventional methods: nearest neighborhood, bilinear, bicubic, cubic, and lanczos4.

The performance of the super-resolution method was evaluated using the mean squared error (MSE), peak signal-to-noise ratio (PSNR), and structural similarity (SSIM) [28] between the high-resolution image and low-resolution and super-resolution images for quantitative evaluation. MSE and PSNR between two images *f* and *g* with m×n pixels are calculated as below:

(1) $$\begin{array}{ccc} \mathrm{MSE} & = & \frac{1}{mn}\sum_{i=0}^{m-1}\sum_{j=0}^{n-1}{\left[f(i,j)-g(i,j)\right]}^{2} \end{array}$$

(2) $$\begin{array}{ccc} \mathrm{PSNR} & = & 10\cdot \log_{10}\left(\frac{255^{2}}{\mathrm{MSE}}\right) \end{array}$$

SSIM is defined as follows:

(3) $$\mathrm{SSIM}=\frac{\left(2\mu_{x}\mu_{y}+C_{1}\right)\left(2\sigma_{xy}+C_{2}\right)}{\left(\mu_{x}^{2}+\mu_{y}^{2}+C_{1}\right)\left(\sigma_{x}^{2}+\sigma_{y}^{2}+C_{2}\right)}$$

where *x* and *y* represent the 7×7 windows in images *f* and *g*, $\mu_{x}$ and $\mu_{y}$ represent the average of *x* and *y*, $\sigma_{x}^{2}$ and $\sigma_{y}^{2}$ represent the variance of *x* and *y*, and $\sigma_{xy}$ represents the covariance of *x* and *y*. $C_{1}$ and $C_{2}$ are variables to stabilize the division with weak denominators. Since we used RGB multi-channel images, these indices were calculated for each channel and average values of the channels were obtained. Lower value of MSE and higher values of PSNR and SSIM indicate a better quality of images.

### 2.3. Performance Evaluation of Disease Classification

Following the processes described in Section 2.2, we generated the low-resolution and super-resolution images for both of training and test images. Next, we conducted disease classification using the high-resolution, low-resolution, and super-resolution images, where we expected that we would achieve better accuracy with the super-resolution images than with the low-resolution ones, because the super-resolution images contained much more information—such as lesions—than the low-resolution images.

We used a convolutional neural network (CNN) for the disease classification. In a training step, 20% of the training images were used for validation. We trained the CNN for 50 epochs with mini batches of size 64.

### 2.4. Architecture of SRCNN

**Figure 1:**
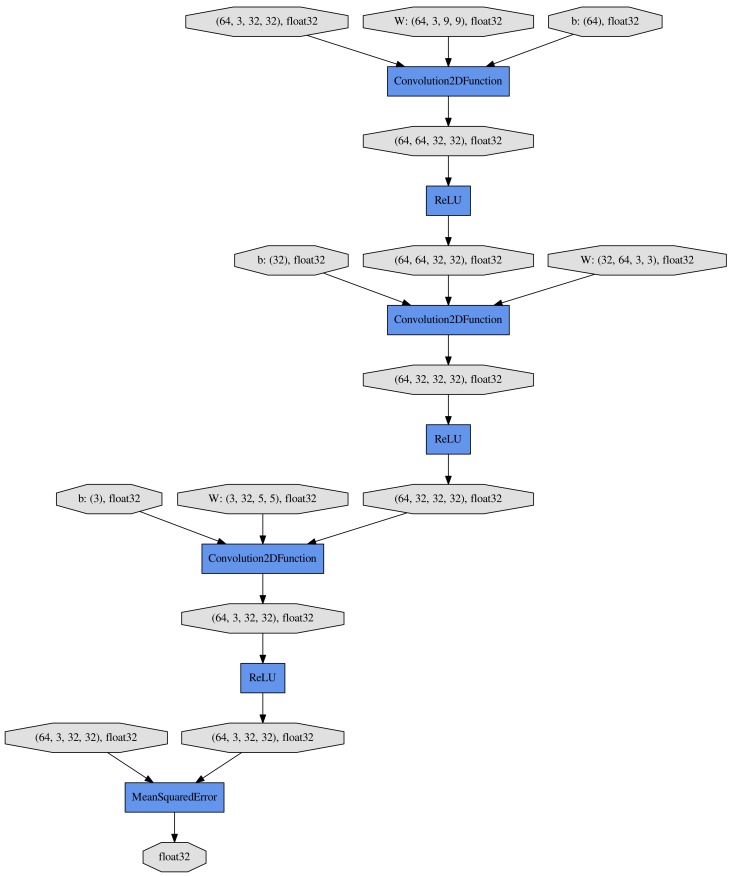
Architecture of the SRCNN model.

The neural network design for this research is shown in Figure 1, which was slightly modified from Dong et al. [19] to avoid the image size reduction, and to produce RGB images directly.

The first layer of the network is a 9 × 9 pixel convolutional layer with a stride of 1 × 1 pixels (in the horizontal and vertical directions) and padding of 4 × 4 pixels (in the horizontal and vertical directions). This convolutional layer maps the RGB in the input image to 64 feature maps using a 9 × 9 pixel kernel function. The second layer is a 3 × 3 pixel convolutional layer with a stride of 1 × 1 pixels and padding of 1 × 1 pixels, mapping the 64 feature maps of the first layer to 32 feature maps. The third layer is a 5 × 5 pixel convolutional layer with a stride of 1 × 1 and padding of 2 × 2 pixels, mapping to 3 feature maps. Rectified linear unit (ReLU) layers are adopted in all the convolutional connected layers. We used MSE as a loss function.

### 2.5. Architecture of CNN for Disease Classification

We used AlexNet architecture [29] for the disease classification. It has five convolutional layers, three subsampling layers, and three fully-connected layers. All the convolutional and fully connected layers are connected with ReLU layers. AlexNet also uses dropout that avoids over-fitting in a training step by reducing complex co-adaptations of neurons [29]. Cross entropy was used as a loss function.

### 2.6. Implementation

All calculations were conducted using Python 2.7 on Ubuntu 14.04 Linux system. All experiments were run on the Amazon Elastic Compute Cloud with single GPU of NVIDIA Tesla K80. The CNN models for super-resolution and disease classification were implemented in Chainer 1.24.0 [30]. Image processing—using procedures such as rescaling, blurring, and patch extraction—was conducted using “scikit-image” and “scikit-learn” packages for Python.

## 3. Results

### 3.1. Super-Resolution

**Figure 2:**
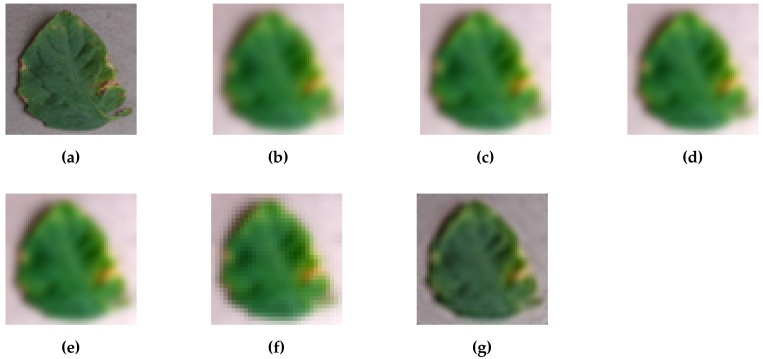
Examples of high-resolution, low-resolution, and super-resolution images. (**a**) Original; (**b**) Bilinear; (**c**) Cubic; (**d**) Bicubic; (**e**) Lanczos; (**f**) NearestNeighbor; (**g**) SRCNN.

An example of results of the super-resolution with magnification scale 4 is shown in Figure 2, where low-resolution images generated by 5 different conventional methods are also shown. First, we can recognize the detailed appearance of a lesion on the leaf in the original image (Figure 2a). On the other hand, in the low-resolution images Figure 2b–f, appearances are completely blurred and the sharpness and smoothness of leaf contour is lost. In the super-resolution image (Figure 2g)—although it is still difficult to recognize the details of the lesion—global characteristics, such as the color of the lesion can be recognized.

**Figure 3:**
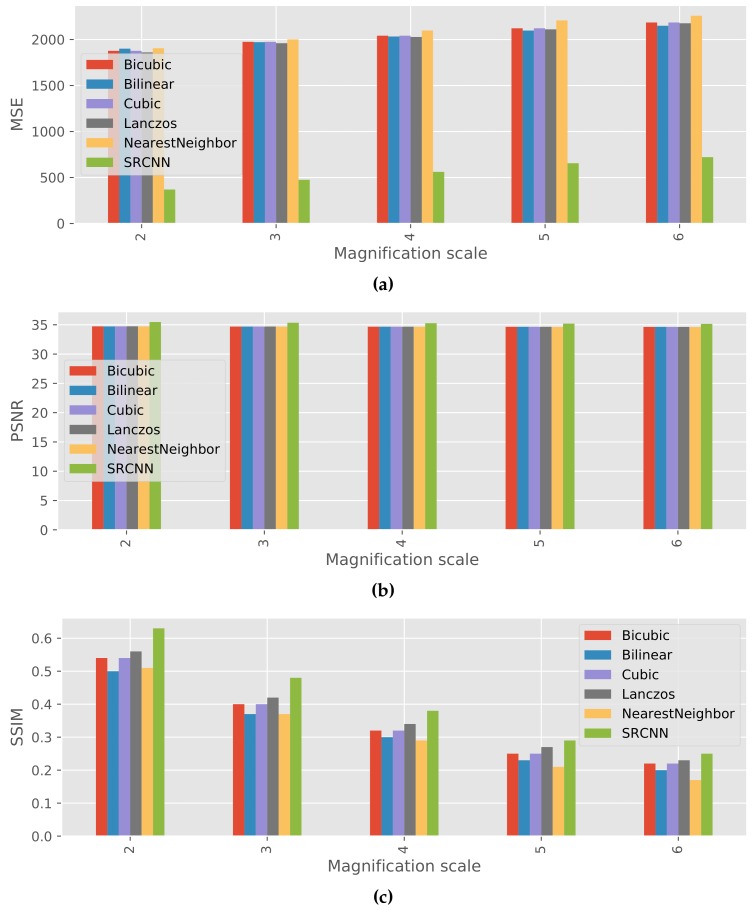
Comparison of average quantitative results produced by MSE, PSNR, and SSIM for different magnification scales. (**a**) MSE; (**b**) PSNR; (**c**) SSIM.

The average results of quantitative evaluation are shown in Figure 3. These results indicate that SRCNN performed the best among all the methods at any magnification scales in terms of MSE, PSNR, and SSIM. In SRCNN, MSE was lower by at least 1428, and PSNR and SSIM were higher by at least 0.51 and 0.02 than other methods.

Although the super-resolution method performed very well, 2% of the test images resulted in higher MSE and lower PSNR and SSIM than the other low-resolution images. The number of test images that failed in super resolution at magnification scales 2–6 were 64, 76, 94, 86, and 61, respectively.

### 3.2. Disease Classification

**Figure 4:**
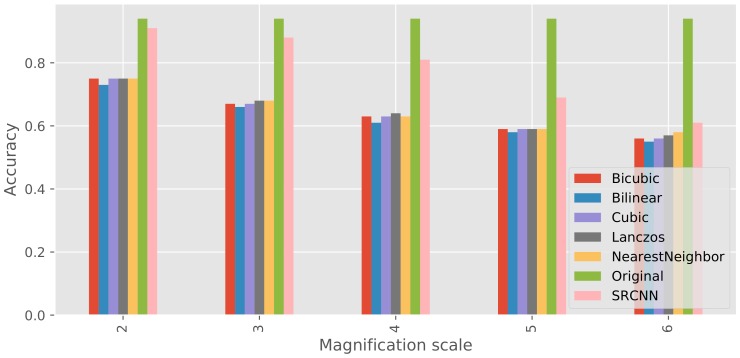
Accuracy of disease classification using high-resolution, low-resolution, and super-resolution images at different magnification scales.

The overall accuracy of disease classification using all kinds of images generated with different magnification scales is shown in Figure 4. The accuracy achieved was the highest when original images were used for testing. The accuracy achieved using super-resolution images was better than that achieved using low-resolution images generated by any kind of methods at all magnification scales.

**Figure 5:**
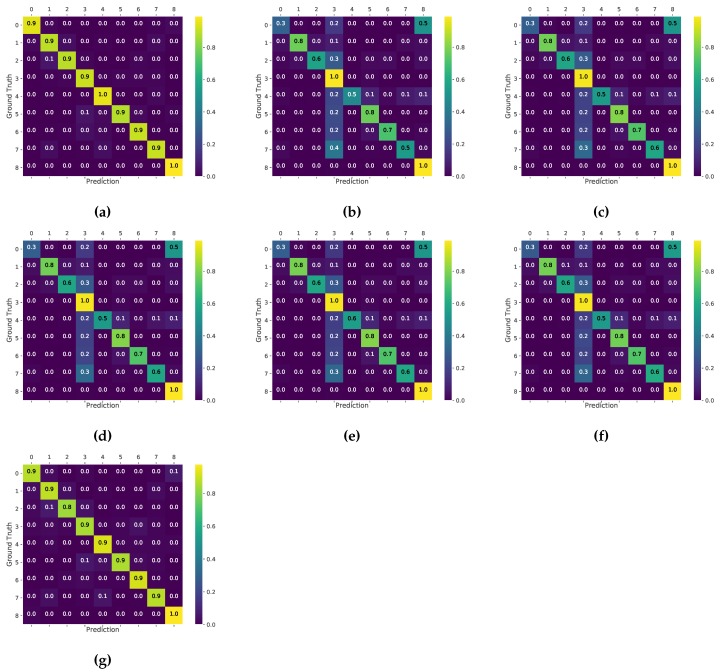
Confusion matrix of disease classification using high-resolution, low-resolution, and super-resolution images with magnification scale 2. Values are normalized by the number of elements in each class. Numbers on x and y axes indicate the ID of diseases in Table 1. (**a**) Original; (**b**) Bilinear; (**c**) Cubic; (**d**) Bicubic; (**e**) Lanczos; (**f**) NearestNeighbor; (**g**) SRCNN.

Confusion matrices of disease classification using high-resolution, low-resolution, and super-resolution images are shown in Figure 5. The confusion matrices are normalized to 0–1 by the number of elements in each class. In the high-resolution and super-resolution images, classifications were almost perfect in all classes. On the other hand, the result of low-resolution images indicated low accuracy in some of the classes, particularly in Xanthomonas campestris pv. Vesicatoria and *Tetranychus urticae*.

## 4. Discussion

This study investigated a super-resolution method for increasing the spatial resolution of disease images of tomato plants. The super-resolution was applied at different magnification scales. We also evaluated the effectiveness of the super-resolution images for further analysis—for instance, disease classification where we expected that classification accuracy with super-resolution images would be better than that with low-resolution ones, because the super-resolution images convey much more information—such as lesions—than the low-resolution images. For the super-resolution, we used a state-of-the-art method based on deep learning, SRCNN.

The result of qualitative evaluations showed that images produced by SRCNN were clearer and less blurred than the low-resolution images generated by 5 different conventional methods. It means that the SRCNN was able to restore the information which was in the original images but was lost in the low-resolution images. The result of quantitative evaluation demonstrated the result of qualitative evaluations: MSE from original images was lower and PSNR and SSIM were higher than those for the low-resolution images at all magnification scales.

In terms of processing time, the super-resolution method takes more than the conventional methods we used to generate the low-resolution images. Roughly speaking, the SRCNN took 3 h to finish 50 epochs in the training process, and 1 s to generate one high-resolution image in the testing process. On the other hand, the conventional methods require no training process, and took less than a second for image generation. Although SRCNN training takes a long time, it needs to be conducted only once, and the generated model can be used for the images taken at different cultivation fields. In addition, state-of-the-art devices such as Jetson (NVIDIA, Santa Clara, CA, USA) will enable to not only the shortening the time needed for image generation in SRCNN, but also processing in real-time on UAV in the near future.

Additionally, SRCNN outperformed the other conventional methods in disease classification by a large margin at magnification scales 2–5. Especially at magnification scales 2–3, we achieved almost the same accuracy as with the original image. At magnification scale 6, the classification accuracy was slightly improved (≥+0.03), but it was very much less than that at the smaller magnification scales. These results indicate that our approach not only recovers the information lost in low-resolution images but also has a good influence on further image analysis, which has not been objectively evaluated in the previous similar studies. Proposed approaches will accelerate image-based phenotyping and vigor diagnosis in the field, because they allow the flight of a drone at high altitudes to capture a crop in a cultivation field, but secure the image accuracy for further analysis. One of the drawbacks of the proposed method is the training data collection. Even though such data collection requires time and labor costs, long-term costs should be less than those incurred using drones at low altitude to capture high-resolution crop images.

The failure of super-resolution occurred for about 2% of the test images. Some of the failures were due to unexpected objects, mainly the finger of the photographer, in an image. One of other possible causes of the failures was the use of MSE as a loss function. According to Ledig et al. [31], the use of MSE lost high-frequency details such as texture, because it produces pixel-wise averages of plausible solutions which are typically over-smoothed. Super-resolution generative adversarial network (SRGAN) proposed by Ledig et al. [31] overcomes this issue by using loss calculated on feature maps of CNN, which are more invariant to changes in pixel space than MSE. In future study, we need to compare SRGAN with SRCNN for the purpose of disease classification.

The accuracy of disease classification showed that the use of low-resolution images resulted in lower accuracy than that of high-resolution images for any diseases. Especially, the accuracy of Xanthomonas campestris pv. Vesicatoria was greatly decreased because of many misclassification between it and *Tomato yellow leaf curl virus*. It seems to be caused by the similar appearances of lesion of the diseases: both of Xanthomonas campestris pv. Vesicatoria and *Tomato yellow leaf curl virus* turn the leaf color into yellow (Figure A1) On the other hand, in the super-resolution image, the accuracy was improved and almost equal to the accuracy of the high-resolution image. These results indicate that super-resolution method reconstructed the detailed appearance of lesions and enabled the identification of the diseases.

In this study, we used disease images acquired from ground cameras instead of UAV-based cameras. However, as shown in Figure A1, most of the images in the Plant Village dataset were taken from above a leaf, which are similar to images taken by UAV-based cameras. Although we believe that our approach is effective with UAV-based cameras, it is necessary to evaluate our approach with images taken by UAV-based cameras for practical applications in future studies.

One of the important techniques in deep learning, including CNN, is transfer learning, also as known as fine-tuning. In this research, we slightly modified the network architecture from the original one proposed by Dong et al. [19] to avoid the image size reduction, and to produce RGB images directly. We therefore could not use their pre-trained weights for fine-tuning. Even though fine-tuning was not conducted, we have already demonstrated the superiority of SRCNN to other conventional methods. However, it is necessary to conduct the training of the networks using a large image dataset such as ImageNet [32] beforehand and to evaluate their performance in future studies.

## 5. Conclusions

We proposed an approach using a super-resolution method to accelerate the image-based phenotyping and vigor diagnosis in agriculture. First, we applied a super-resolution method based on deep convolutional neural networks—SRCNN—to low-resolution images of nine kinds of tomato diseases. The result showed that SRCNN outperformed conventional image scaling methods in term of MSE, PSNR, and SSIM. Next, we conducted CNN-based classification of the diseases images using high-resolution, low-resolution, and super-resolution images. The resulting classification accuracy was better with super-resolution images than with low-resolution images. Some misclassifications because of similar appearances between diseases occurred with low-resolution images, but were improved with super-resolution images. These results indicated that super-resolution methods reconstructed the detailed appearance of lesions, and enabled the identification of the diseases. Our approach has the potential to accelerate phenotyping and vigor diagnosis using UAVs and image analysis technologies.

## Figures and Tables

**Table 1:** The number of images of each tomato disease in Plant Village dataset [27].

ID	Disease	Num.
0	Xanthomonas campestris pv. Vesicatoria	2127
1	*Alternaria solani*	2579
2	*Phytophthora infestans*	1910
3	*Septoria lycopersici*	1771
4	*Tetranychus urticae*	1676
5	Tomato mosaic virus	373
6	*Fulvia fulva*	952
7	*Corynespora cassiicola*	1404
8	*Tomato yellow leaf curl virus*	5357

## Supplementary Materials

The Plant Village dataset is currently unavailable online but will be available soon. Please refer to https://plantvillage.org/posts/6948-plantvillage-dataset-download.

## Abbreviations

The following abbreviations are used in this manuscript:

SRCNN	Super resolution convolutional neural network
CNN	Convolutional neural network
MSE	Mean squared error
PSNR	Peak signal-to-noise ration
SSIM	Structural similarity
